# Sestrin2 Overexpression Ameliorates Endoplasmic Reticulum Stress-Induced Apoptosis via Inhibiting mTOR Pathway in HepG2 Cells

**DOI:** 10.1155/2022/2009753

**Published:** 2022-12-10

**Authors:** Huiling Hu, Zhijun Luo, Xiuli Liu, Lisi Huang, Xiaoxia Lu, Rui Ding, Chaohui Duan, Yuqing He

**Affiliations:** ^1^Department of Clinical Laboratory, Sun Yat-Sen Memorial Hospital, Sun Yat-Sen University, Guangzhou, China; ^2^Guangdong Provincial Key Laboratory of Malignant Tumor Epigenetics and Gene Regulation, Sun Yat-Sen Memorial Hospital, Sun Yat-Sen University, Guangzhou, China; ^3^Department of Emergency, The Seventh Affiliated Hospital, Southern Medical University, Foshan, Guangdong 528244, China; ^4^Translational Medicine Research Center, Zhujiang Hospital, Southern Medical University, Guangzhou, Guangdong 510515, China

## Abstract

Sestrin2 is a highly conserved stress-inducible protein, acting as a crucial part in regulating homeostasis in response to various stress conditions in the cell. However, the role of Sestrin2 in regulating cell apoptosis related to endoplasmic reticulum (ER) has not been fully investigated. Our study presented here aims to reveal the effect of Sestrin2 in tunicamycin (TM)-induced cell apoptosis related to ER stress and its underlying molecular mechanisms. The results demonstrated that Sestrin2 expression was significantly upregulated correlated with ER stress responses in TM treated HepG2 cells. Sestrin2 overexpression obviously alleviated ER stress with the determination of ER stress-related proteins expression. In addition, Sestrin2 overexpression inhibited cell apoptosis with the examination of apoptosis-related proteins and TUNEL assay. However, Sestrin2 knockdown further promoted the ER stress-mediated cell apoptosis. The further mechanistic study revealed that Sestrin2 overexpression inhibited TM-induced mTOR pathway activation. Taken together, our current study indicated that Sestrin2 overexpression ameliorates ER stress-induced apoptosis via inhibiting mTOR pathway in HepG2 cells.

## 1. Introduction

The liver is a highly complex organ with multiple metabolic functions including transamination, gluconeogenesis, lipogenesis, bile, and protein synthesis [1]. The liver disease remains a major public health problem worldwide, with incidence increasing with each passing decade. In China, approximately 300 million people are afflicted with major liver diseases, including viral hepatitis, alcoholic liver disease, and nonalcoholic fatty liver disease (NAFLD). Globally, more than 2 million people die of liver diseases each year, accounting for about 4% of all deaths [2, 3]. Liver injury, induced by overdosed acetaminophen (APAP), alcohol ingestion, hepatitis virus, insulin resistance, and NAFLD, is the common pathological basis of various liver diseases [4–7]. Among those risk factors provoking liver injury, APAP overdosage may induce hepatocellular necrosis and cytolysis, leading to acute liver failure. Also, excessive alcohol ingestion causes hepatocyte injury through oxidative stress, which is linked to the production of ROS and inhibition of the expression of antioxidant enzymes by ethanol [8–10]. However, despite extensive exploration of the pathophysiology of liver diseases, there is still no effective targeted therapy regimen. Therefore, it is necessary to explore the underlying mechanism of liver injury and seek out potential therapeutic targets.

Hepatocytes are the main functional cells of the liver, accounting for about 70% of all cells in the normal liver, and play important roles in regulating homeostasis, regeneration, and injury recovery [11]. As the main region of protein synthesis, hepatocytes contains abundant endoplasmic reticulum (ER), an organelle which takes charge of protein assembly, protein glycosylation, disulfide conformation adjustment, and cellular calcium storage, allowing this cell type be sensitive to changes of intracellular homeostasis [6, 12, 13]. If alcohol triggers oxidative stress, misfolded proteins accumulate in the ER, or other damage factors exists, ER will initiate unfolding protein response (UPR) to facilitate repair of tissue damage and restore homeostasis. However, if hepatocytes fail to adapt to the stressor, then excessive or prolonged UPR activation, namely, ER stress [14], leads to the development of liver injury through hepatocyte apoptosis [15], steatosis [16], and fibrosis [17]. ER stress consists of a complex network of signal transduction pathways from ER to the nucleus and is initiated by three ER transmembrane proteins, namely, PERK, ATF6, and IRE1, which keeps inactive under physiological conditions. When ER stress occurs, these transmembrane proteins are activated sequentially. Activated PERK induces phosphorylation of eukaryotic initiation factor 2*α* (eIF2*α*) and then promotes the translation of transcription factor ATF4. By regulating the expression of protein synthesis (e.g., ER chaperones GRP78 and GRP94), degrading protein aggregates and initiates autophagy, and PERK/ATF4 pathway helps to restore ER homeostasis and promotes survival. But sustained ER stress promotes apoptosis through ATF4/CHOP pathway. Similarly, activated ATF6 and IRE1 help ER restore function by regulating the expression of another transcription factor XBP1 or otherwise result in ER stress associated apoptosis through IRE1/JNK and ATF6/CHOP pathway [6, 12, 13]. Besides, ER stress also activates apoptosis via apoptotic-related genes (Bax and Bcl-2) and an apoptotic caspase cascade in which Caspase-12, Caspase-3, and Caspase-9 are involved [18, 19]. Previous studies showed that ER stress plays an important role in liver injury through up-regulating expression of apoptotic protein such as Caspase-3, Caspase-12, JNK, and CHOP, along with the increase of GRP78, ATF6, and XBP1, which are hall markers of ER stress [12, 13]. Moreover, numerous studies have shown that through these signal pathway, ER stress results in various liver diseases, including viral hepatitis, alcoholic liver disease (ALD), NAFLD, insulin resistance, and ischemia-reperfusion injury [6, 20].

Sestrin2 is a member of the sestrin family, also known as a highly conserved stress-inducible protein, induced by kinds of stress conditions, including DNA damage, oxidative stress, ER stress, and metabolic stress [21, 22]. Numerous research studies have demonstrated that Sestrin2 expression can be upregulated under ER stress conditions and exert cytoprotective functions [22, 23]. Han et al. found that Sestrin2 expression is upregulated in cholestatic liver injury. Once induced, Sestrin2 in turn protects hepatocyte from cholestasis-induced injury by ameliorating hepatic ER stress [24]. In addition, a study conducted by Li et al. revealed that overexpression of sestrin2 limits ER stress, promoting neuronal survival and improving functional recovery after spinal cord injury [25]. All these studies indicated that Sestrin2 might serve as an important regulator that maintains regulating cellular homeostasis under excessive ER stress. Therefore, understanding how Sestrin2 is regulated with different stress conditions is very helpful for us to study Sestrin2.

The mammalian target of rapamycin (mTOR) is one of the five components which make up rapamycin complex 1 (mTORC1). As a master growth regulator and an autophagy inhibitor, mTOR plays a crucial part in controlling cell proliferation, metabolism, and autophagy through perception of change of amino acids, hormones, and growth factors. Amino acids, especially leucine, as one of critical environmental stimulator, are quite important in mTORC1 activation [26]. In addition, Sestrin2, playing the role of leucine sensor, regulates mTORC1 pathway by sensing the change of leucine through its affinity for leucine by structural change [27]. Recently, Gao et al. demonstrated that kinsenoside alleviates alcoholic liver injury by inhibiting ER stress via AMPK/mTOR pathway [28]. Accumulating studies have demonstrated that mTOR hyperactivity takes a pivotal role in regulating fatty acid synthesis, hepatic insulin resistance, and type 2 diabetes [29, 30]. Several mTOR signaling pathways are involved in the development of liver injury, including the LKB1/AMPK/mTOR metabolic axis, NF-*κ*B/mTOR signaling, and TSC/mTOR signaling. The team of Zhou et al. discovered that Puerarin inhibits hepatocellular apoptosis in rats with 2-AAF/PH-induced liver injury through inhibition of mTOR signal pathway by up-regulating the expression of p-mTOR [31]. Gao et al. demonstrated that kinsenoside can reverse the ethanol-induced inhibition of the AMPK in mice model with ethanol/CCl4-induced alcoholic liver injury through LKB1/AMPK/mTOR pathway and leads to the inhibiting of phosphorylation of mTOR, which thereby activates autophagy to remove damaged proteins [28]. Considering that Sestrin2 is an upstream regulator of mTOR and closely related to ER stress, in the present study, we try to investigate whether Sestrin2 is involved in the regulation of ER stress-induced hepatocyte apoptosis through mTOR pathway.

In this study, we found Sestrin2 overexpression ameliorates ER stress-induced apoptosis via inhibiting mTOR pathway in HepG2 cells. These results indicated the potential target of Sestrin2 for modulating the mTOR signaling pathway to alleviate apoptosis in response to cell stress in liver injury. Understanding the precise functions and the underlying mechanism of Sestrin2 in regulating cellular homeostasis will provide the evidence for future experimental studies and facilitate the development of novel therapeutic strategies for liver diseases.

## 2. Materials and Methods

### 2.1. Antibodies and Reagents

We bought primary antibodies of GRP78 (Cat#48119), CHOP (SAB 40744) from Signalway Antibody (USA). The primary antibodies of XBP1 (Cat#24868-1-AP), BCL2 (Cat#12789-1-AP), BAX (Cat#50599-2-IG), Caspase3 (Cat#19677-1-AP), Caspase12 (Cat#24868-1-AP), Cytochrome C (Cat# 66264-1-IG), were *β*-Actin (Cat# 66009-1-IG) were supplied by Proteintech (China). We purchased the primary antibodies from Cell Signaling Technology (USA), and the antibodies include cleaved Caspase3 (Cat#9661s), Caspase3 (Cat#14220S) mTOR (Cat#2983s), p-mTOR ser2448 (Cat#5536s), 4E-BP1 (Cat#9644), p-4E-BP1 (Cat#2855), P70S6K (Cat#9202), and p-P70S6K (Cat#9205). ATF6 (Cat#WL02407) was from Wanleibio (China). We performed the study by using secondary antibodies of HRP-conjugated Goat anti-Rabbit IgG (Cat#HS101-01) and HRP-conjugated Goat anti-mouse IgG (Cat#HS201-01). These antibodies were supplied by TransGEN Biotech (China). Sangon Biotech (China) provided us with Tunicamycin (Cat#A611129-0005).

### 2.2. Cell Culture

HepG2 was cultured in DMEM medium, supplemented with 10% FBS and 1% penicillin/streptomycin. We applied varying concentration of TM (0, 0.5, 1, 2, 5, and 10 *μ*g/mL, dissolved in DMSO) to induce ER stress in HepG2 cells. The dose of TM was decided according to previous studies with minor modifications [32]. Concentration of 5 *μ*g/mL of TM was selected for future analysis.

### 2.3. Cell Viability Test

We performed cell viability tests with Cell Counting Kit (CCK-8, TargetMol, USA). 96-well plate was incubated with 100 *μ*L cell for 24 h, and the process followed the instruction of the suppliers. We added 10 *μ*L CCK-8 directly in culture medium in each well after treatment with various concentration of TM for 24 h. Then, the incubation process lasted for 1 h with the temperature of 37°C. We exerted absorbance test for multiple times with Synergy TM H1 multidetection microplate reader (Bio-tek Instruments Inc., USA) at 450 nm. Cell viability was tested by the results of absorbance. The 100% viable parameter was obtained from the cells in control medium.

### 2.4. Transient Transfection

For Sestrin2 overexpression and knockdown experiments, the plasmids of pcDNA3.1-Sestrin2, and pLKO.1-shSestrin2 and corresponding control plasmids were synthesized from Tsingke Biotechnology (Guangzhou, China). The sequences used for shSestrin2 were shown in Supplementary Table 1. The transfection was performed with ViaFect reagent (Vigorous Biotechnology, Beijing, China) according to the manufacturer's instructions. Tissue culture plate with 6-well was used for cells culturing. After 24 h, the culture medium was replaced prior to transfection. The dosages of vector and plasmid were 2 *μ*L and 5 *μ*g for one well. We incubated the cell for 12 h, and then treatment of TM for 24 h was applied. The sample was ready for analysis such as mRNA and protein expression test.

### 2.5. Western Blot

The HepG2 cells were lysed with radio-immunoprecipitation assay (RIPA) buffer (Fd Bioscience, Hangzhou, China) supplemented with protease/phosphatase inhibitor cocktail (Fd Bioscience) on ice for 30 min and centrifuged at 12000 rmp at 4°C for 5 min. Then, BCA protein assay kit (Beyotime, Shanghai, China) was used to detect the concentration of protein. The equal amounts (30 *μ*g/lane) of total protein were separated using a 12% SDS-PAGE gel and transferred to PVDF membranes. The membranes were blocked in 1 × TBST with 5% (*w*/*v*) skim milk or 1 × TBST with 5% *w*/*v* bovine serum albumin (BSA) at room temperature. To ensure the specificity of primary antibodies, antibodies specificity and the molecular weight of target proteins were tested with HepG2 cells extracts first of all (Supplementary Figure 1). Afterwards, the whole PVDF membranes were cut into several rectangular bands for incubation of corresponding primary antibodies according to the molecular weight of the target protein under the direction of the protein ladder, and then the membranes were finally scanned using MP ChemiDoc imaging system (Bio-Rad, USA). The relative expression of the proteins was normalized to that of *β*-Actin.

### 2.6. Quantitative Real-Time PCR (qRT-PCR)

We extracted RNAs from HepG2 cells with Trizol reagent (Invitrogen) following with the instructions of manufacturer. The integrity and concentration of RNA were evaluated by NanoDrop One ultraviolet spectrophotometer (Thermo Fisher Science, USA). We reversely transcribed RNA into cDNA with the reverse transcription reagent kit (Applied Biological Materials, Vancouver, Canada) following with the instructions of suppliers. SYBR Green Master Mix (Roche Life Science, Basel, Switzerland) was used in the LightCycleer 480 system (Roche) to perform the qPCR test. Conditions were set to initial 95°C for 5 min, then 45 cycles with the same pattern, that is 10 s on 95°C, 15 s on 60°C and 20 s on 72°C. We ran the melting routine immediately after the last cycle. To exclude primer dimers' influence, the routine is held for 10 s at 4°C and read every 1.0°C. These experiments were tested in triplicate. We used the 2^−ΔΔCt^ method to standardize the relative abundance of mRNAs with *β*-Actin mRNA as the invariant control. The following primers were used: Sestrin2-F: 5′-TCTTACCTGGTAGGCTCCCAC-3′, Sestrin2-R: 5′-AGCAACTTGTTGATCTCGCTG-3′,*β*-Actin-F: 5′- CGCCGCCAGCTCACCATGGAT-3′,*β*-Actin-R: 5′- CCACCATCACGCCCTGGTGC-3′.

### 2.7. TdT Mediated dUTP Nick End Labeling (TUNEL) Staining

We applied the TUNEL test by TUNEL FITC Apoptosis Detection Kit (Vazyme Biotech, Shanghai, China). Concentration of 0.01% poly-L-Lysine-coated glass coverslips was used to plate HepG2 cells in 24-well tissue culture plates. PBS was used to rinse the slides after the treatment and then fixed the slides with cold 4% paraformaldehyde (PFA) in PBS for 25 min with temperature of 4°C. Subsequently, we rinsed the slides twice for 5 min with PBS. PBS was used to permeabilize the cells with 0.2% Triton X-100 for 5 min. Finally, we used equilibration buffer to incubate the cells for 20 min at 25°C. In a humidified and dark environment, TNNEL reaction mixture was added at 37°C, and counterstaining with DAPI (Invitrogen). A fluorescence microscope (DMI 8, Leica) was used to evaluate the sections.

### 2.8. Statistical Analysis

Data were presented as mean ± standard error of the mean (SEM). Statistical analyses were processed with the SPSS 23 software (IBM Corp., Armonk, NY, USA) and GraphPad Prism 8 (GraphPad Software Inc., San Diego, CA, USA). Unpaired Student's *t* test was performed to evaluate the significance of the difference between two groups. Comparisons of means for multiple treatment groups against control group were conducted using one-way ANOVA followed by Dunnett's 2-sided*post-hoc* test. For the correlation analysis between the protein levels of Sestrin2 and apoptotic-related markers, the Spearman's rank-order method was used for all tests since most of the variables were non-normally distributed. A *p*-value of less than 0.05 was considered statistically significant. Details about statistical analyses were given with the concerning tests in the figure legends.

## 3. Result

### 3.1. Sestrin2 Expression Was Significantly Up-Regulated Correlated with ER Stress Responses in TM Treated HepG2 Cells

**Figure 1:**
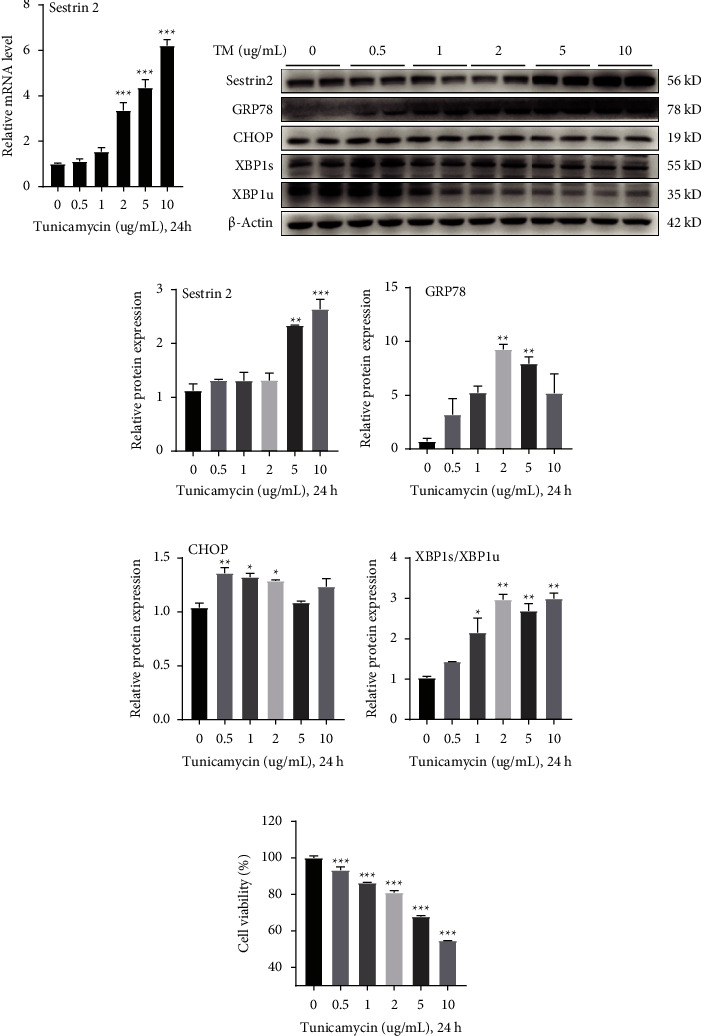
TM treatment-induced Sestrin2 up-regulation and ER stress. (a) mRNA level of Sestrin2 treated with various concentrations of TM (one-way ANOVA, Dunnett t post hoc test; *F*_(5, 18)_ = 7.62, *P* < 0.001). (b) Representative western-blot results show Sestrin2 expression and proteins involving ER stress, such as GRP78, CHOP, XBP1s, and XBP1u. (c–f) Western blot quantitative analysis of Sestrin2 and proteins involving ER stress (one-way ANOVA, Dunnett *t* post hoc test; for Sestrin2: *F*_(5, 6)_ = 27.27, *P* < 0.001; for GRP78: *F*_(5, 6)_ = 8.97, *P*=0.009; for CHOP: *F*_(5, 6)_ = 9.12, *P*=0.009; for XBP1s/XBP1u: *F*_(5, 6)_ = 20.27, *P*=0.001). (g) Viability of HepG2 cells with different TM concentrations treatment (one-way ANOVA, Dunnett t post hoc test; *F*_(5, 18)_ = 291.97, *P* < 0.001). ^*∗*^*P* < 0.05, ^∗∗^*P* < 0.01, ^∗∗∗^*P* < 0.001.

TM, as a natural antibiotic, induces endoplasmic reticulum (ER) stress by inhibiting glycosylation of newly synthesized proteins in the ER leading to the disruption of their maturation [33–35]. In order to study the effect of ER stress induced by TM on Sestrin2 expression, HepG2 cells were treated with varying concentrations of TM from 0 to 10 *μ*g/mL. qPCR and western blot analysis showed that Tm increased Sestrin2 levels in a concentration-dependent manner (Figures 1(a)–1(c)). In addition, the protein expression of ER stress markers, such as the GRP78, CHOP, and XBP1s/XBP1u was significantly upregulated (Figures 1(b), 1(d)–1(f)). The data indicated that Sestrin2 expression correlates with ER stress responses in TM-treated HepG2 cells. To further eliminate the possibility of unwanted cytotoxicity effects caused by excessive concentration of TM, we performed a cell viability assay to assess its efficacy at the preferred doses. The results indicated that with the concentration of TM increasing, the cell viability was decreased dramatically; as expected, higher TM treatment (10 *μ*g/mL) resulted in significant destructive effects on cell viability compared with 5 *μ*g/mL TM treated group (Figure 1(g)). Taken together, considering the TM at a dose of 5 *μ*g/mL yielded obviously ER stress induction with limited adverse effects, we propose the TM dose of 5 *μ*g/mL, which was a preference choice when to investigate the effect of ER stress-induced HepG2 cell apoptosis.

### 3.2. Overexpression of Sestrin2 Ameliorates the TM-Induced ER-Stress

**Figure 2:**
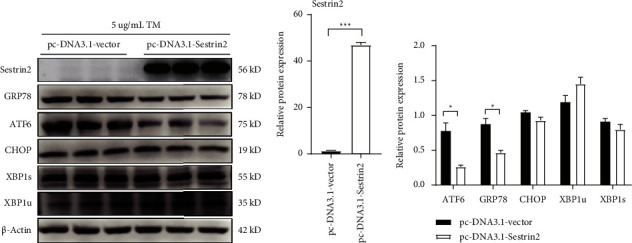
Sestrin2 overexpression ameliorates TM-induced ER stress. (a) Representative western-blot results of Sestrin2 and ER stress-related proteins, ATF6, GRP78, CHOP, XBP1u and XBP1s in the indicated groups. (b) Western blot quantitative analysis of Sestrin2 (unpaired two-tailed*t*-test; *t*_(4)_ = −38.44, *P* < 0.001). (c) Western blot quantitative analysis of ER stress-related proteins (unpaired two-tailed*t*-test; for ATF6: *t*_(4)_ = 4.32, *P* = 0.012; for GRP78: *t*_(4)_ = 4.54, *P*=0.010). ^*∗*^*P* < 0.05, ^∗∗∗^*P* < 0.001.

Firstly, in order to study the effect of Sestrin2 overexpression on ER stress induced by TM, we transfected HepG2 with pcDNA3.1-Sestrin2 or control plasmid and followed with 5 *μ*g/mL TM treatment. The Sestrin2 expression and ER stress biomarkers, such as the GRP78, ATF6, CHOP, XBP1s, and XBP1u were examined with western blot analysis. With the comparison to the control group, Sestrin2 was obviously overexpressed in the pcDNA3.1-Sestrin2 group (Figure 2). What is more is that Sestrin2 overexpression ameliorates the TM-induced ER stress, as well as the ER stress-related proteins expression, for example, GRP78 and ATF6 (Figure 2).

### 3.3. Overexpression of Sestrin2 Ameliorates Tunicamycin-Induced HepG2 Cells Apoptosis

**Figure 3:**
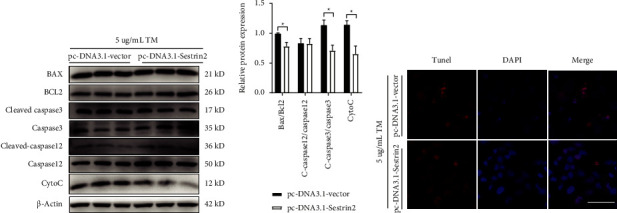
Sestrin2 overexpression ameliorates TM-induced HepG2 cells apoptosis. (a) Representative western-blot results of proteins involved with cell apoptosis (b) Quantitative analysis of proteins involved with cell apoptosis (unpaired two-tailed*t*-test; for Bax/Bcl2: *t*_(4)_ = 3.03, *P*=0.039; for C-caspase3/caspase3: *t*_(4)_ = 3.33, *P*=0.029; for CytoC: *t*_(4)_ = 3.19, *P*=0.033). (c) Representative images of TUNEL positive HepG2 cells in indicated groups. Scale bar, 50 *μ*m. ^*∗*^*P* < 0.05.

To examine the detail function of Sestrin2 on TM-induced HepG2 cells apoptosis, cell apoptosis-related proteins, such as BAX, BCL2, Cleaved-caspase3, Caspase3, Cleaved-caspase12, Caspase12, and CytoC were detected with western-blot assay after overexpression of Sestrin2. In addition, we performed a TUNEL assay to test HepG2 cells apoptosis. The results demonstrated that the expression of BAX/BCL2, Cleaved-caspase3/Caspase3, and CytoC was inhibited in the Sestrin2 overexpression group (Figures 3(a) and 3(b)). Furthermore, compared to the control group, TUNEL-positive cells were obviously decreased in the Sestrin2 overexpression group (Figure 3(c)). All these findings indicated that overexpression of Sestrin2 significantly alleviates the apoptosis of HepG2 cells.

### 3.4. Sestrin2 Knockdown Promotes Tunicamycin-Induced HepG2 Cells Apoptosis

**Figure 4:**
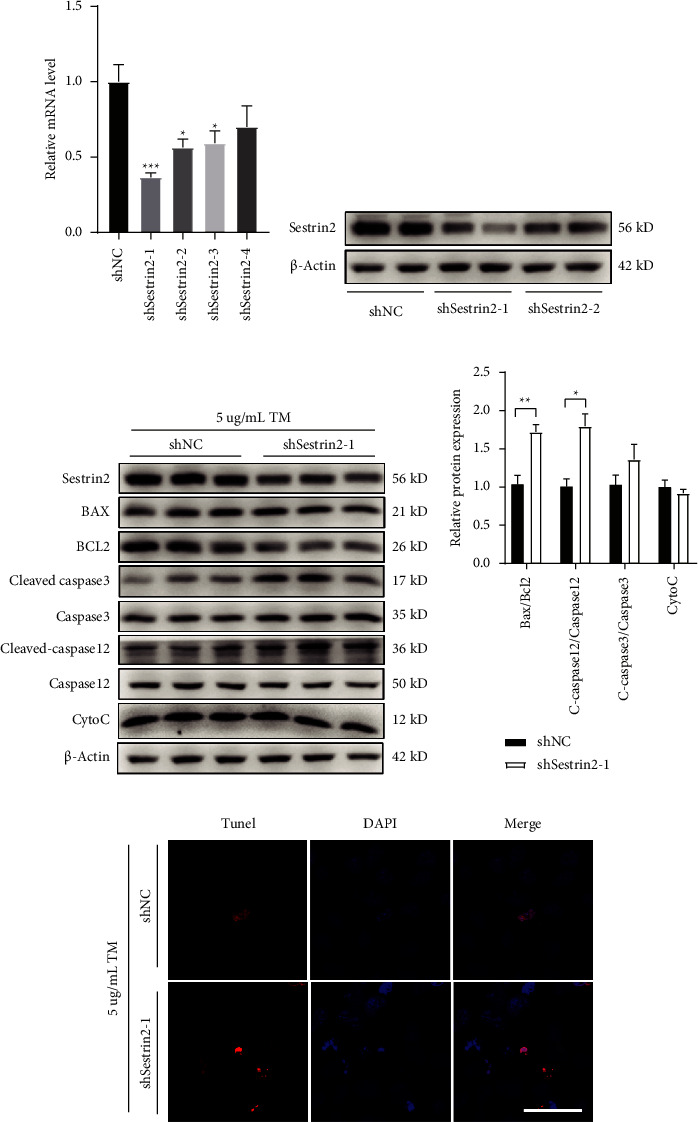
Sestrin2 knockdown promotes TM-induced HepG2 cells apoptosis. (a) shRNA-mediated silencing of Sestrin2 in HepG2 cells was confirmed by qPCR analysis (one-way ANOVA, Dunnett *t* post hoc test; *F*_(4, 15)_ = 6.34, *P*=0.003). (b) Western blot analysis further validating Sestrin2 knockdown in HepG2 cells as determined by protein bands presented at 56 kDa. (c) Representative Western blot results of proteins involved with cell apoptosis. (d) Quantitative analysis of proteins involved with cell apoptosis (unpaired two-tailed*t*-test; for Bax/Bcl2: *t*_(4)_ = −4.61, *P*=0.010; for C-caspase3/caspase3: *t*_(4)_ = −4.15, *P*=0.014). (e) Representative images of TUNEL positive HepG2 cells in indicated groups. Scale bar, 50 *μ*m. ^*∗*^*P* < 0.05, ^∗∗^*P* < 0.01, ^∗∗∗^*P* < 0.001.

To further determine the potential role of Sestrin2 inhibition on ER stress-induced apoptosis. Sestrin2 knockdown was performed using Sestrin2-targeted shRNA cloned into the plasmid. HepG2 cells were transfected with either the shRNA vector control plasmid (shNC) or the Sestrin2 targeting shRNA plasmid (shSestrin2) for 24 h. qPCR analysis revealed that Sestrin2 mRNA expression was decreased to 36.7% by shSestrin2-1 (*P* < 0.001), 56.4% by shSestrin2-2 (*P*=0.015), 76% by shSestrin2-3 (*P*=0.023), and 70.1% by shSestrin2-4 (*P*=0.113) separately compared to the shNC, among which shSestrin2-1 showed significant suppression of Sestrin2 mRNA and protein levels (Figures 4(a) and 4(b)). Afterwards, HepG2 cells were transfected with either shNC or shSestrin2 plasmid for 12 h and then treated with tunicamycin for 12 h. Compared with shNC groups, there was a significant increase in the expression of BAX/BCL2 (*P* < 0.001) and Cleaved-caspase12/Caspase12 (*P*=0.014) (Figures 4(c) and 4(d)). In addition, TUNEL assay confirmed that Sestrin2 knockdown obviously increased TUNEL-positive cells (Figure 4(e)). All these findings indicated that Sestrin2 inhibition significantly promotes the apoptosis of HepG2 cells.

### 3.5. The Expression Level of Sestrin2 was Negatively Correlated with That of Apoptotic-Related Markers in HepG2 Cells

**Figure 5:**
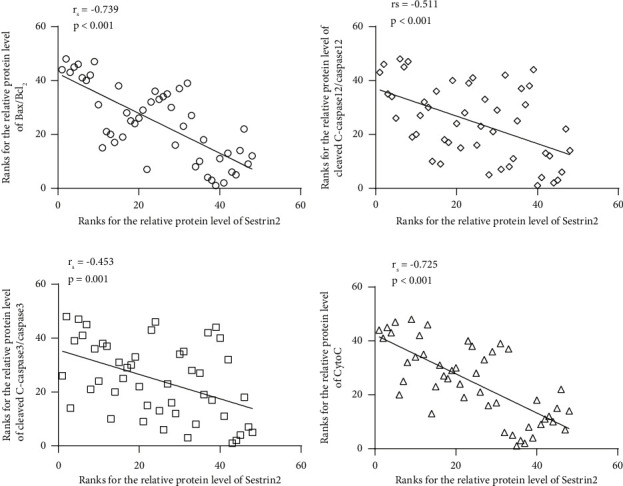
The expression level of Sestrin2 was negatively correlated with that of apoptotic-related markers in HepG2 cells. This graphic plotted the rank correlation between the relative protein levels of Sestrin2 and (a) Bax/Bcl2 (*ρ* = −0.739, *P* < 0.001), (b) C-caspase12/Caspase12 (*ρ* = −0.453, *P*=0.001), (c) C-caspase3/Caspase3 (*ρ* = −0.511, *P* < 0.001) and (d) CytoC (*ρ* = −0.725, *P* < 0.001) in HepG2 cells in which the gene expression of Sestrin2 was manipulated to be either upregulated or down-regulated by plasmid transfection. Spearman's two-tailed correlation analysis was used to compare the fold change data obtained from Western Blot densitometry analysis of indicated proteins. On the subplot, each point represents an individual sample of HepG2 cell culture (*n* = 48 from 5 independent experiments), and no outlier was excluded from this study. The solid line is the best fit through the data, with the respective Spearman's correlation coefficient displayed as rs.

In order to check the reliability of the inhibiting potential of Sestrin2 in ER stress-induced apoptosis, the Sestrin2 gene expression was reprogrammed in HepG2 cells by transient transfection of either pcDNA3.1-Sestrin2 or shSestrin2 plasmids. The protein abundance of Sestrin2 and apoptotic-related markers was detected in a total of 48 individual samples of HepG2 cell culture from 5 independent experiments using western blot analysis. The bands obtained were quantified using densitometry analysis by ImageJ, and then Spearman's rank-order correlation analysis was used to compare the fold change of the normalized expression of indicated proteins. Intriguingly, we found strong negative correlations between the protein levels of Sestrin2 and apoptotic-related markers, including Bax/Bcl2 (Spearman's rho = −0.739, *P* < 0.001, Figure 5(a)), C-caspase12/Caspase12 (Spearman's rho = −0.453, *P*=0.001, Figure 5(b)), C-caspase3/Caspase3 (Spearman's rho = −0.511, *P* < 0.001, Figure 5(c)), and CytoC (Spearman's rho = −0.725, *P* < 0.001, Figure 5(d)). These results further corroborated our finding that Sestrin2 may play an important role in regulating ER stress-induced apoptosis in HepG2 cells.

### 3.6. Sestrin2 Overexpression Inhibits TM-Induced mTOR Pathway Activation

**Figure 6:**
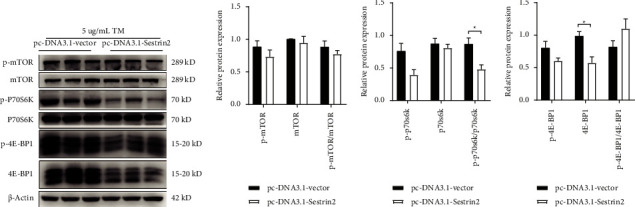
Sestrin2 overexpression inhibits TM-induced mTOR pathway activation. (a) Representative western-blot results of major proteins associated with mTOR pathway. (b) Statistical analysis of p-mTOR, mTOR and their ratio (unpaired two-tailed *t*-test; all *P* values >0.05). (c) Statistical results of p-P70S6K, P70S6K and their ratio (unpaired two-tailed *t*-test; for p-p70s6k/p70s6k: *t*_(4)_ = 3.30, *P*=0.030). (d) Quantitative results of p-4E-BP1, 4E-BP1 and their ratio (unpaired two-tailed *t*-test; for 4E-BP1: *t*_(4)_ = 3.62, *P*=0.022). ^*∗*^*P* < 0.05.

We then research the potential mechanism of the inhibition of Sestrin2 overexpression on ER stress induced by TM and cell apoptosis. Expression of mTOR, P70S6K, and 4E-BP1 and their phosphorylated morphology were tested for their roles as mTOR pathway related proteins. The outcome proved that mTOR, 4E-BP1, and p-P70S6K/P70S6 expression was dramatically inhibited in the pcDNA3.1-Sestrin2 group (Figure 6). The data altogether demonstrated that Sestrin2 overexpression inhibits TM-induced mTOR pathway activation. We propose that Sestrin2 may inhibit TM-induced ER stress and cell apoptosis through mTOR pathway.

## 4. Discussion

ER stress is the main cause of liver injury [36], and the molecular mechanism of ER stress is complex. Sestrin2 exerts as an important part in the process of ER stress, but its action pathway is not fully elucidated. In our study, we observed that TM could induce ER stress in HepG2 cells, and Sestrin2 expression was elevated under ER stress. Sestrin2 overexpression obviously alleviated TM-induced ER stress and cell apoptosis. Further mechanistic study revealed that Sestrin2 overexpression inhibited TM-induced mTOR pathway activation. In conclusion, we propose that Sestrin2 overexpression ameliorates ER stress-induced apoptosis via inhibiting mTOR pathway in HepG2 cells. This study demonstrated the potential target of Sestrin2/mTOR pathway for the treatment of cell apoptosis associated with ER stress.

Tunicamycin is a bacterial toxin which activates mammalian cells' UPR by blocking of N-linked glycosylation [37]. It causes ER stress in hepatocytes through the UPR [38]. By detecting the marker proteins of ER stress, we noticed the ER stress induce by TM treatment, and the expression of Sestrin2 was significantly increased. This is consistent with previous research findings [32]. Interestingly, GRP78 and CHOP expression increased first and decreased gradually while the TM concentration elevated. According to previous reports, GRP78 and CHOP are the important biomarkers of ER stress [38, 39], and Sestrin2 protects against bavachin-induced ER stress [40]. So, we proposed that a high concentration of TM treatment leads to overexpression of Sestrin2, and overexpression of Sestrin2 reversely inhibited the expression of GRP78 and CHOP, which eventually suppressed the ER stress.

To test this hypothesis, Sestrin2 was overexpressed with transfected with pcDNA3.1-Sestrin2 plasmid in HepG2 cells. By detecting the marker proteins of ER stress, we found that the expression of ER stress-related proteins was significantly inhibited after overexpression of Sestrin2. The outcomes altogether are in accordance with previous studies, in which Park et al. demonstrated that Sestrin2 has an important regulatory role in obesity-induced ER stress [23], and Yang et al. showed that Sestrin2 can alleviate bavachin-induced ER Stress [40].

Autophagy is modulated by ER stress, and this process induces cell survival or death. However, the interaction between them is still obscured [41]. One of the primary pathogenesis in the liver disease is apoptosis, and autophagic activity modulated the hepatic apoptosis. Hepatic apoptosis is regulated by autophagic activity. Park et al. found that elevated Sestrin2 attenuates obesity-induced liver apoptosis [23]. What is more is that Sesn2 inhibits APAP-induced hepatocyte death [42]. CytoC, BAX, and BCL2 are recognized as biomarkers of apoptosis [43, 44]. In our studies, by detecting the expression of the proteins mentioned above, as well as the TUNEL positive cells, we proved that Sestrin2 overexpression can reduce TM-induced apoptosis in HepG2 cells.

mTOR, as a serine/threonine kinase, modulates numerous basic processes in cell, such as protein synthesis, growth, metabolism, aging, regeneration, autophagy [45]. Yang et al. have found that Sestrin2 regulated the BV-induced ER stress through the signaling pathway of AMPK/mTORC1 [40]. Han et al. demonstrated that Sestrin2 deficiency resulted in hepatic ER stress induced by cholestasis, but overexpression of Sestrin2 ameliorated ER stress induced by bile acid [24]. Therefore, to investigate the systematic process of the Sestrin2 inhibition on ER stress induced by TM and cell apoptosis, the main protein expression involved in the mTOR pathway was evaluated, and the results indicated that Sestrin2 overexpression inhibits TM-induced mTOR pathway activation. We propose that Sestrin2 may inhibit TM-induced ER stress and cell apoptosis through mTOR pathway.

However, further validation should be conducted in this study, such as the mTOR activators should be used when the Sestrin2 was overexpressed to test whether mTOR activation could suppress the protective effect of Sestrin2. Furthermore, in vivo experiments could be performed to validate the inhibition effect of Sestrin2 on liver injury.

## 5. Conclusions

These findings indicate that Sestrin2 overexpression ameliorates ER stress-induced apoptosis via inhibiting mTOR pathway in HepG2 cells. Sestrin2 might be a therapeutic target with potential for modulating the signaling pathway of mTOR to alleviate apoptosis in response to cell stress in liver injury.

## Data Availability

The research data presented in the paper are available from the corresponding authors with reasonable request.

## Supplementary Materials

Supplementary Materials Supplementary Figure 1: The primary antibodies specificity and the molecular weight of target proteins. Supplementary Table 1: The sequence used for shSestrin2.
