# Supplementary material for: Sestrin2 Overexpression Ameliorates Endoplasmic Reticulum Stress-Induced Apoptosis via Inhibiting mTOR Pathway in HepG2 Cells
Source: Int J Endocrinol. 2022 Dec 10;2022:2009753. doi: 10.1155/2022/2009753 (PMC9759384; doi:10.1155/2022/2009753)
Supplement: Supplementary Materials — Supplementary Figure 1: The primary antibodies specificity and the molecular weight of target proteins. Supplementary Table 1: The sequence used for shSestrin2. [file 2009753.f1.docx]

**Supplementary Information for**

**Sestrin2 overexpression ameliorates endoplasmic reticulum stress-induced apoptosis via inhibiting mTOR pathway in HepG2 cells**

Huiling Hu^1,2,*^, Zhijun Luo^3,*^, Xiuli Liu^1,2,*^, Lisi Huang, Xiaoxia Lu^1,2^, Rui Ding^1,2,#^, Chaohui Duan^1,2,#^, Yuqing He^1,2,#^

^*^These authors contributed equally to this work.

^#^ To whom correspondence should be:

Rui Ding, E-mail: dingr6@mail.sysu.edu.cn,

Chaohui Duan, E-mail: duanchh@mail.sysu.edu.cn

Yuqing He, E-mail: heyuqingm@163.com

This file includes:

Supplementary Fig. 1

Supplementary table 1


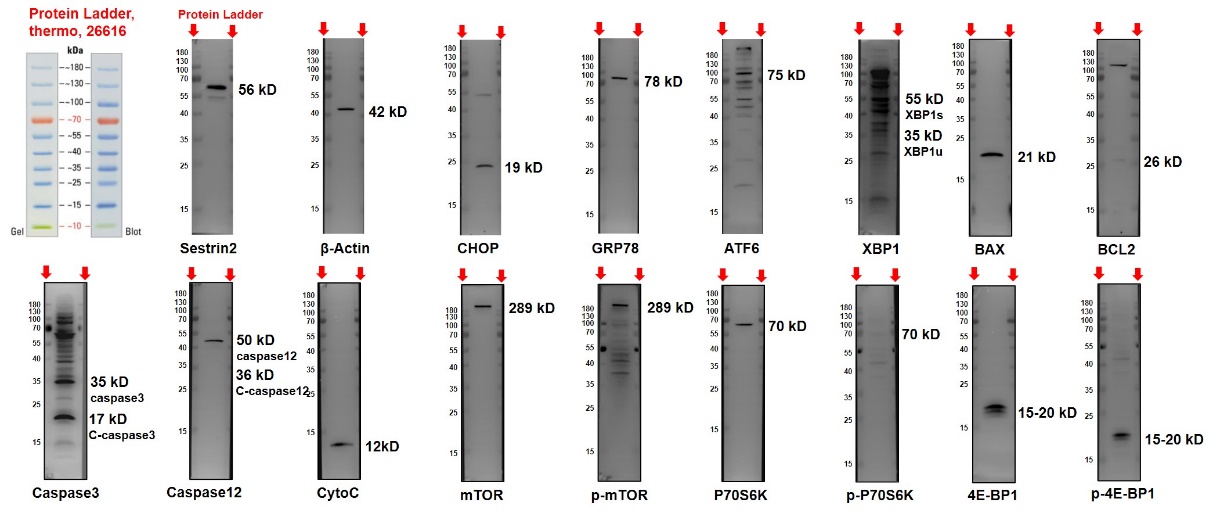


Supplementary Fig. 1. The primary antibodies specificity and the molecular weight of target proteins were tested with HepG2 cells extracts under the direction of the protein ladder.

Supplementary Table 1. The sequence used for shSestrin2

|  | **sequence (5′–3′)** |
| --- | --- |
| shNC | CTTACGCTGAGTACTTCGA |
| shSestrin2-1 | GAAGACCCTACTTTCGGAT |
| shSestrin2-2 | GAGATGGAGAGCCGCTTT |
| shSestrin2-3 | CAGACATGCTGTGCTTTGT |
| shSestrin2-4 | CCGAAGAATGTACAACCTCTT |
